# High-Temperature Isothermal Oxidation Behavior of Newly Developed Fe–Cr–Ni Austenite Stainless Steel

**DOI:** 10.3390/ma19071461

**Published:** 2026-04-05

**Authors:** Mohammed Nawaz Husain, Thangam Muniyandi, Bhuvaneshuwari Balaguru, Kamalan Kirubaharan Amirtharaj Mosas, Ashok Raja Chandrasekar, Dinesh Kumar Devarajan

**Affiliations:** 1 Centre for Nanoscience and Nanotechnology, Sathyabama Institute of Science and Technology, Jeppiaar Nagar, Rajiv Gandhi Salai, Chennai 600119, India; nawazmohammed3718@gmail.com (M.N.H.); thangamtamil89@gmail.com (T.M.); bhuvibalaguru29@gmail.com (B.B.); 2 Advanced Characterization Facility, Centre for Advanced Studies, Sathyabama Institute of Science and Technology, Jeppiaar Nagar, Rajiv Gandhi Salai, Chennai 600119, India; 3 Coating Department, FunGlass—Centre for Functional and Surface Functionalized Glass, Alexander Dubcek University of Trencin, 91150 Trencin, Slovakia; kamalankiruba@gmail.com (K.K.A.M.); ashokraja.chandrasekar@tnuni.sk (A.R.C.)

**Keywords:** stainless steel, isothermal oxidation behavior, parabolic rate of oxidation, major spallation, stress-induced crack

## Abstract

One of the significant causes of failure in aerospace engine components is high-temperature oxidation. Therefore, it is necessary to investigate the high-temperature oxidation behavior of newly fabricated structural materials for aerospace components. From this perspective, the isothermal oxidation behavior and kinetics of newly developed stainless steel (SS) 08X14H were investigated at 750, 950 and 1050 °C for up to 100 h in an air environment. The weight results demonstrate that oxidation in 08X14H increases with time and temperature and follows a parabolic rate law. Major spallation was observed in samples oxidized for 100 and 24 h at 950 °C and 1050 °C, respectively. Structural and morphological analysis of oxidized samples through X-ray diffraction (XRD) and field emission scanning electron microscopy (FESEM) of the surface and cross section reveal the phases present and their distribution. The structural results confirm the formation of Fe_2_O_3_, Cr_2_O_3_, FeCr_2_O_4_ and intermediate (Cr, Fe)_2_O_3_ oxides in the oxidized samples. Surface morphologies reveal that the formation of a Cr_2_O_3_ layer effectively protects the material from further oxidation. At higher temperatures, the coarsening of Fe_2_O_3_ oxides takes place, which leads to the formation of loose and porous oxide scale with stress-induced cracks. The spallation of the outermost Fe_2_O_3_-rich oxide scale was observed, and the matrix is exposed during the extreme oxidation at 950 and 1050 °C for 100 and 50 h, respectively. The cross-sectional morphologies and elemental mapping results reveal a duplex oxide layer with an outermost Fe_2_O_3_ layer followed by an underlying layer of Cr_2_O_3_, (Cr, Fe)_2_O_3_ and FeCr_2_O_4_ spinel beneath the Fe_2_O_3_ layer.

## 1. Introduction

Aerospace components exposed to high temperatures are prone to numerous deformations, including oxidation [1,2], creep [3], fatigue [4] and corrosion [5,6,7], which eventually lead to the components’ failure during service. The materials developed for high-temperature applications should have superior mechanical properties with resistance to deformations [8]. Austenitic SSs constitute a very large steel class in terms of alloys that have been utilized in high-temperature aircraft components such as roller bearings [9], landing gear doors [10], exhaust systems and other crucial parts due to their excellent mechanical, oxidation and corrosion resistance properties, among others [6]. Austenitic steels are composed of alloying elements including chromium (Cr) [11], nickel (Ni) [12,13], molybdenum (Mo) [14], silicon (Si) [15], aluminum (Al) [16] and manganese (Mn) [17], in addition to iron (Fe), to achieve resistance against deformations and offer improved performance at higher operating temperature with increased lifetime [18]. The composition of the alloy differs depending on the requirement and nature of operating conditions. To achieve high-temperature oxidation resistance behavior, an increase in alloying elements, including Cr, Al and Mn, should be carried out to ensure the formation of thick, dense, uniform, slow-growing and pore-free self-protecting oxide layers such as chromia (Cr_2_O_3_) [5], alumina (Al_2_O_3_) [19] and silica (SiO_2_) [20,21]. Therefore, these SSs exhibit the desired oxidation and creep resistance without compromising their mechanical properties [22].

Chromium is the primary element in steel as it has a major influence on the enhancement of the oxidation resistance of the material [22,23]. Therefore, it is essential to add adequate Cr to achieve optimal oxidation protection by forming a protective and durable Cr_2_O_3_ layer. Larger Cr content improves the oxidation resistance of materials. Researchers have reported that a minimum of 15% (wt.%) of Cr is essential to form a stable and dense Cr_2_O_3_ layer [23]. Apart from Cr, Si and Al are known for their ability to enhance the oxidation resistance by facilitating the formation of SiO_2_ and Al_2_O_3_ healing layers, which serve as barriers for the outward and inward diffusion of metallic cations and oxygen anions, respectively [24,25]. Evans et al. (1983) [15] analyzed the effect of silicon in alloying additions using six different nitrated 20Cr–25Ni SSs with silicon composition ranging from 0.05 to 2.35 wt.% and identified that better oxidation resistance and decreased rate of oxidation were shown for an intermediate silicon content of 0.92%. The reason for the increased oxidation resistance in the intermediate composition is the development of a silica interlayer between the Cr_2_O_3_ oxide layer and the metal, which acts as a diffusion barrier [15]. Riffard et al. (2004) investigated the breakaway oxidation behavior in Cr_2_O_3_-forming AISI 304 SS and highlighted the significance of the silica sublayer in providing high-temperature oxidation and corrosion resistance [26]. Gu et al. (2020) [27] demonstrated the influence of Al alloying on the oxidation and corrosion resistance of newly developed ZG40Cr20Ni20 heat-resistant steels. The alloy exhibited superior high-temperature oxidation resistance even after oxidation for 480 h at 1100 °C, and the oxidation resistance increased with increasing Al content up to 5.34 (wt.%) [27].

Various researchers have analyzed the isothermal oxidation behavior of stainless steels [28]. It is well known that high-temperature oxidation in austenite steels in air follows a parabolic rate law [29], controlled by solid-state diffusion of one or more species through a compact oxide layer that progressively thickens over time [26]. The passive oxide film continuously grows with an increase in exposure period to reduce the inward diffusion of oxygen species into the matrix [30]. After reaching the threshold limit, the adhesion between the alloy and the oxide layer weakens, resulting in the detachment of the oxide layer from the alloy subsurface [31,32].

In Fe–Cr-Ni systems, higher temperature exposure up to 800–1000 °C favors the development of oxides like hematite (Fe_2_O_3_), chromia (Cr_2_O_3_), (Fe, Cr, Mn)_3_O_4_ spinel and corundum oxides (Cr_2_O_3_–Fe_2_O_3_) [28]. The Fe_2_O_3_ initially forms at a higher rate due to the rapid diffusion of Fe ions from the matrix. Dheeradhada et al. (2011) [33] observed the presence of these phases in the GE–13L ferritic steels when exposed to temperatures up to 1000 °C. They also observed that the Cr volume fraction in corundum oxide increases with an increase in Cr composition [33]. Hou (2010) stated that the initial formation of corundumh oxides is rich in Fe_2_O_3_ and subsequently converts into Cr-rich phases upon increasing exposure time [34]. Fern et al. (1970) developed a 20.4Cr–25.2Ni–0.67 Nb stainless steel, and observed oxide phases like Cr_2_O_3_, Fe_2_O_3_–Cr_2_O_3_ and FeCr_2_O_4_ spinel phases when exposed at 850 °C for 200 h in a CO_2_ environment [35]. Apart from alloying compositions, several parameters like oxidation temperature, exposure period, partial pressure of oxygen [36,37], aging treatment [38] and microstructure [39] play a significant role in the oxidation resistance of the material. Zhiyuan Chen et al. (2014) [40] studied the effect of partial pressure of oxygen (33.3, 66.6 and 100% of O_2_) in the initial oxidation behavior at 600–950 °C and the stress generated in the oxide scale in SS 441. In lower partial pressures like 33.3 and 66.6%, linear oxidation kinetics were observed and the oxide layer consisted of Fe-containing oxides. Also, it was observed that the stress growth in the oxide scale increases with an increase in the partial pressure of oxygen [40]. K. C. Tripathl et al. (1970) investigated the effect of grain size on the oxidation resistance of 20Cr–25Ni–Nb SS and discovered that better oxidation resistance was observed in larger grain size microstructures compared to fine grain ones [41]. The application of most of the austenite stainless steels is limited to 950 °C due to the volatization of chromia above 1000 °C, which leads to spallation of the oxide layer and development of stress-induced cracks, ultimately resulting in material failure. Additionally, information regarding the high-temperature oxidation resistance of these alloys at elevated temperatures remain limited. Therefore, it is essential to investigate the isothermal high-temperature oxidation behavior of newly developed alloys under such conditions. In this context, the isothermal high-temperature oxidation behavior of 08X14H stainless steel was investigated at 750, 950 and 1050 °C to evaluate its oxidation resistance before its application in structural components, thereby reducing the risk of component failure during service.

The stainless steel 08X14H is a newly developed wrought Fe–Cr-Ni-based austenite SS with alloying additions such as Cr, Ni, Mo, Si, Mn and Cu. In this work, the isothermal oxidation behavior of wrought 08X14H SS is investigated in air at high temperatures such as 750, 950 and 1050 °C for exposure periods of 1, 24, 50 and 100 h. Furthermore, this work seeks to explore the oxidation kinetics of 08X14H SS at higher temperatures along with the structural, compositional and morphological changes of oxides using various analytical techniques.

## 2. Experimental Setup

### 2.1. Materials and Standard Heat Treatment Conditions

The 08X14H SS was procured from Mishra Dhatu Nigam Limited (MIDHANI, Hyderabad, India) as a rod with a 16 mm diameter and 120 mm length. The chemical composition of the as-cast 08X14H SS is given in Table 1.

The solution treatment and aging procedure of the as-cast 08X14H were performed in an inert argon atmosphere. The solution treatment was performed at 1300 °C for 5.5 h and isothermally aged for 2.5 h at 1050 °C, followed by sub-zero treatment [42,43]. Tempering of the aged 08X14H was conducted at 300 °C for 5 h, and finally air-quenched to room temperature.

### 2.2. Sample Preparation

For the high-temperature oxidation experiments, disc-shaped samples of 3 mm thickness and 15 mm diameter were sliced using the wire EDM process. The specimen surfaces were polished using emery sheets of various grit sizes (80, 220, 400, 600, 800, 1000, 1200, 1500 and 2000) using the Struers Tegramin 25 polishing machine (Struers, Ballerup, Denmark). A mirror finish was achieved by fine polishing using Struers’s MD–MOL cloth suspended with 9 µm diamond solution. The surface contamination was removed by sonicating the fine polished samples using a bath ultrasonicator with acetone and ethanol.

### 2.3. Oxidation Experiments

To examine the high-temperature isothermal oxidation behavior of 08X14H SS in an air environment, isothermal oxidation experiments were carried out at various temperatures, 750, 950 and 1050 °C, for different exposure durations of 1, 24, 50 and 100 h. The isothermal oxidation experiments were performed in an interchangeable box-type high-temperature furnace with a maximum temperature of 1400 °C, manufactured by VB ceramics (Chennai, India).

### 2.4. Characterization Techniques

An analytical balance with a precision of 1 × 10^−5^ g was used to weigh the oxidized specimens both before and after the oxidation experiments in order to examine the weight changes during isothermal oxidation. The structural analysis of the oxidized samples was carried out using an X-ray diffractometer (XRD, Thermo Scientific–ARL Equinox 3000, Waltham, MA, USA) through Cu Kα1 radiation (wavelength = 1.54 Å). A field emission scanning electron microscope (FESEM, Carl Zeiss Sigma 300, Oberkochen, Germany) equipped with energy dispersive X-ray spectroscopy (EDS, Oxford, Abingdon, UK) was used to examine the oxide morphologies and their distribution. The thickness of the oxidized layers was investigated on the cross-sectional area. The oxidized samples were hot-moulded vertically with bakelite powder, and metallographically polished for cross-sectional analysis.

## 3. Results and Discussion

### 3.1. Isothermal Oxidation Behavior Analysis by Weight Gain Curves

**Figure 1:**
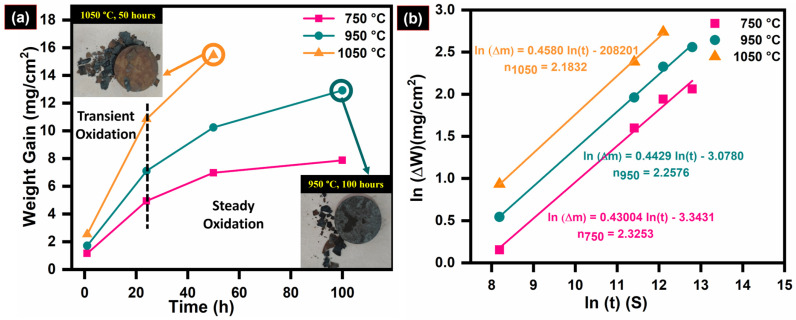
(**a**) Weight gain curves and (**b**) ln (mg/A) graph showing the oxidation behavior of 08X14H SS oxidized in an air environment.

The weight change behavior of the 08X14H austenite SS oxidized at various temperatures was measured for each testing temperature using the following Equation (1), and the resultant graphs are shown in Figure 1a,b.

(1) $${\left(∆W\right)}^{n}=kt$$

where ∆*W* is the weight gain or loss per unit area (∆m/A) during oxidation in mg·cm^−2^, *n* is the kinetic parameter which signifies the kinetic model of the behavior of 08X14H during oxidation, *k* is the constant representing the rate of oxidation, and *t* is the time in seconds.

From the graph (Figure 1a), it can be seen that the higher weight gain in 08X14H was observed at the lower exposure time (1 and 24 h), which decreased with higher duration (50 and 100 h). This pattern of weight gain corresponds to the parabolic rate law of oxidation. The oxidation rate in 08X14H was found to be 4.26 × 10^−4^, 9.61 × 10^−4^ and 2.12 × 10^−3^ mg·cm^−2^h^−1/2^ at 750, 950 and 1050 °C, respectively. Their corresponding oxidation coefficients (n) of 2.32, 2.25 and 2.18 confirm that the isothermal oxidation kinetics in 08X14H follow the parabolic rate.

At 750 °C, the steel subsurface was covered by a gray-colored oxide layer during the initial exposure period (1 and 24 h), which became black with higher exposure duration (50 and 100 h) at 750 °C. After 50 h of exposure at 950 °C, minor oxide scale spallation was detected. After 100 h of exposure at 950 °C, major spallation of the oxide scale from the steel subsurface was observed. In the case of oxidation at 1050 °C, major spallation of the oxide layer occurred within 24 h of exposure and complete spallation was observed after 50 h of exposure.

### 3.2. Structural Analysis of High-Temperature Oxidized 08X14H SS

**Figure 2:**
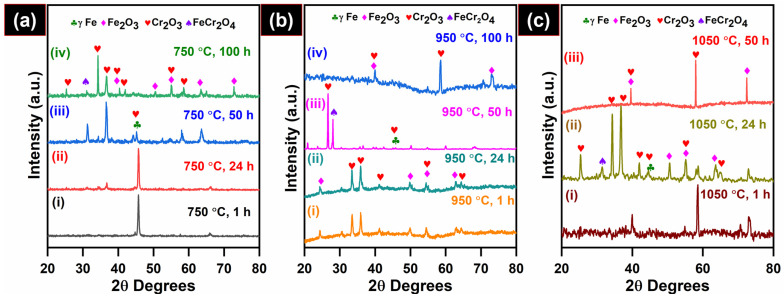
XRD spectra of 08X14H samples oxidized in an air environment at (**a**) 750 °C, (**b**) 950 °C, and (**c**) 1050 °C and exposure times of 1, 24, 50 and 100 h.

Figure 2 shows the XRD spectral results of isothermal oxidized 08X14H at 750, 950 and 1050 °C for different exposure durations. The diffraction pattern revealed the presence of phases including Fe_2_O_3_ (JCPDS; 00-024-0072), Cr_2_O_3_ (JCPDS; 00-001-1294) and FeCr_2_O_4_ spinel (JCPDS; 01-089-2618). The structural information between Fe_2_O_3_ and (Cr, Fe)_2_O_3_ is quite similar, as both phases have similar cell parameters [44]. Therefore, (Fe, Cr)_2_O_3_ peaks are not indexed in the XRD spectra. The spectral results demonstrated that both oxidation temperature and exposure time were found to strongly influence the oxidation behavior of 08X14H steels.

After 1 h of oxidation at 750 °C, no oxide-related peaks were detected, and the XRD spectra (Figure 2a, spectra (i)) were dominated by the reflections from the austenite Fe matrix corresponding to the (1 1 1) and (2 0 0) planes at 2θ = 43.83 and 64.46°, respectively. After 24 h of exposure, slight Cr_2_O_3_ and Fe_2_O_3_ peaks appear in the XRD spectra (Figure 2a, spectra (ii)), indicating the early nucleation of oxide phases over the subsurface. With prolonged oxidation up to 50 and 100 h (Figure 2a, spectra (iii) and (iv)), the γ–Fe peaks were significantly decreased, while the oxide peaks of Fe_2_O_3_ and Cr_2_O_3_ phases at 37.3° and 33.68° became predominant.

At 950 °C, the matrix peaks are dominated by Fe_2_O_3_ and Cr_2_O_3_ oxide peaks in the specimens oxidized for 1 h (Figure 2b, spectra (i)). In contrast to the behavior at 750 °C, the γ–Fe peaks remained dominant for up to 24 h (Figure 2b, spectra (ii)). The rapid suppression in the intensity of the γ–Fe peaks within 1 h at 950 °C clearly demonstrates that the oxidation rate was significant. This observation is consistent with the increased oxidation rate observed in the weight gain curves at 950 °C. On 50 h of exposure at 950 °C (Figure 2b, spectra (iii)), enhanced intensities of Cr_2_O_3_ peaks, along with a reduction in Fe_2_O_3_ peak intensity, were observed, which could obviously be due to the outer Fe_2_O_3_ layer spallation. This spallation was also confirmed by the surface morphologies analyzed using FESEM (after 50 h of oxidation at 950 °C). Following 100 h of oxidation (Figure 2b, spectra (iv)), a single dominant peak appeared at 55.69° corresponding to Cr_2_O_3_, indicating complete spallation of the Fe_2_O_3_ outer layer and direct exposure of the underlying Cr_2_O_3_, (Cr, Fe)_2_O_3_ and spinel (Cr, Fe)_3_O_4_ layers. At 1050 °C, the oxidation rate was faster, with the Cr_2_O_3_ peak reaching an intensity comparable to that of Fe_2_O_3_ after 1 h of exposure (Figure 2c, spectra (i)), indicating rapid oxide formation and minor spallation compared with oxidation at 750 and 950 °C. After 24 h of exposure (Figure 2c, spectra (ii)), the oxide scale was completely detached, leading to the pronounced appearance of Cr_2_O_3_ phases. The XRD pattern after 50 h (Figure 2c, spectra (iii)) revealed re-nucleation and growth of the Fe_2_O_3_ oxides on the alloy subsurface following the spallation of the earlier-formed scale, confirming re-oxidation of the exposed surface.

### 3.3. Morphological and Compositional Analysis of Isothermal Oxidized 08X14H SS at 750–1050 °C in Air

**Figure 3:**
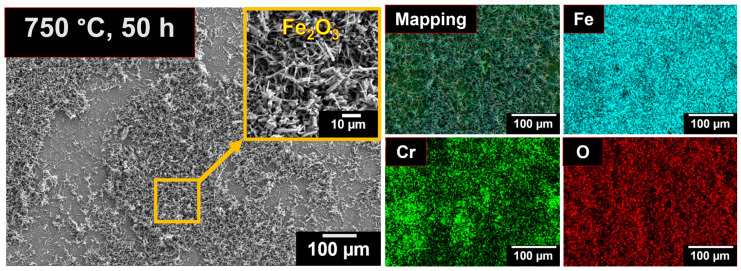
Surface morphologies of 08X14H oxidized in an air environment at 750 °C for 50 h in an air atmosphere and their corresponding EDS elemental mapping.

**Figure 4:**
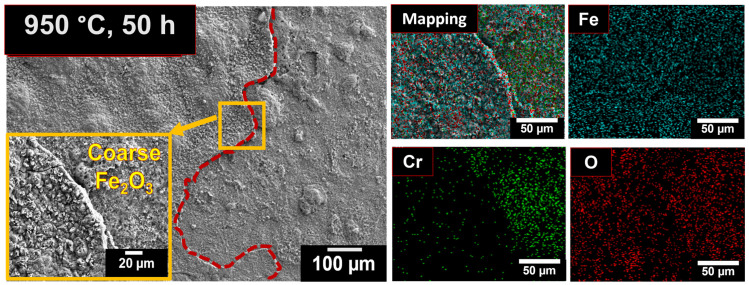
Surface morphologies of 08X14H oxidized in air at 950 °C for 50 h, along with corresponding EDS elemental mapping.

**Figure 5:**
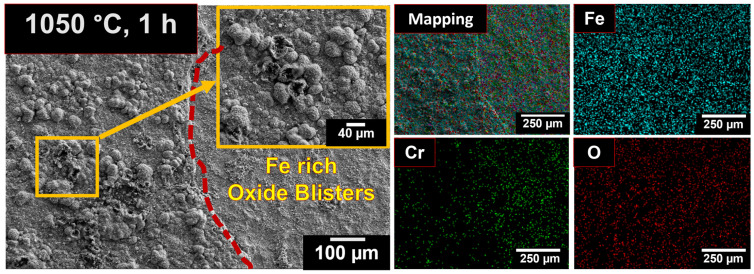
Surface morphologies of 08X14H after oxidation in air at 1050 °C, after 1 h of exposure, with the corresponding elemental maps.

**Figure 6:**
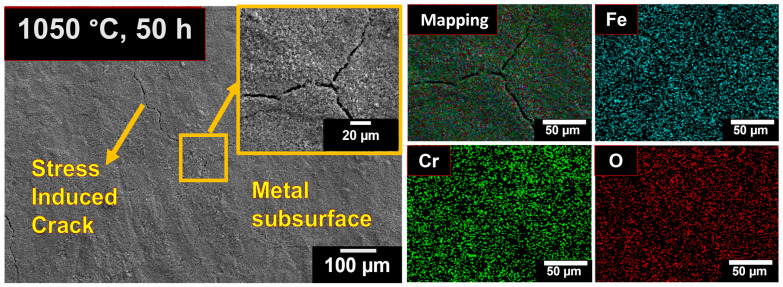
Surface micrographs of 08X14H oxidized in air at 1050 °C for 50 h, with their respective elemental maps.

Figure 3, Figure 4, Figure 5 and Figure 6 show the FESEM micrographs to demonstrate the effect of oxidation on the surface at various temperatures with respect to the change in time. After 1 h at 750 °C, the nucleation of the oxide nodules enriched with Cr and Fe was evident on the surface. The primary nucleated oxides were identified as Cr_2_O_3_ and Fe_2_O_3_, which formed even at low oxygen partial pressures, as indicated by the Ellingham diagram [45], and remain stable at elevated temperatures. As oxidation progresses, these nuclei grow into a continuous oxide film as the oxidation time proceeds.

During 50 h of exposure at 750 °C, a discontinuous Fe_2_O_3_ layer was formed on the surface. A mixed alveolar or lamellar and sprangled morphology was exhibited in the Fe_2_O_3_ layers. Similar distribution for the hematite layer was observed by Salem et al. (2025) during the investigation of oxidation during thermal fatigue damage in AISI H11 hot work tool steel [46]. During the prolonged exposure up to 100 h, a continuous Fe_2_O_3_ layer was developed on the alloy subsurface. These oxide layers were dense, uniform, and free from pores or voids, providing effective protection against further oxidation. The observed saturation in weight gain during extended oxidation was due to the development of this protective layer (Figure 1a). Elemental mapping indicated that the oxide scale was predominantly rich in Fe, along with the noticeable existence of Cr. However, despite the significant Cr content within the oxides, a distinct Cr_2_O_3_ layer was not formed after 50 h of exposure at 750 °C.

Oxidation at 950 °C occurs more rapidly compared to at 750 °C due to the higher thermal energy, which enhances oxygen diffusivity and provides sufficient activation energy at elevated temperatures [2]. Within 1 h of exposure at 950 °C, a continuous Fe_2_O_3_ oxide layer was observed. However, after prolonged exposure of 50 h at 950 °C the oxide layer became coarse, leading to minor spallation of the oxide scale. At the spallation sites, an additional underlying layer enriched in Cr and Fe was observed, which was identified as (Fe, Cr)_2_O_3_ and Cr_2_O_3_. Similar oxide formation [13,19] beneath the Fe_2_O_3_ layer has been reported in earlier studies [24,33]. With continued exposure, the granular oxides coarsen further into spinel-type structures. After 100 h of exposure, complete spallation of the oxide layer occurs from the alloy subsurface, and the corresponding fractograph is shown in Figure 1a.

During oxidation at 1050 °C for 1 h, minor spallation of the oxide layer was found with the formation of oxide blisters. These blisters consisted of lamellar grains and were enriched in Fe. Similar oxide blister formation has been reported by Stott et al. (1989) during investigations into the high-temperature isothermal oxidation behavior of 310 and 321 SSs [47]. At specific sites, these oxide blisters were found to be cracked, and voids were also observed. New oxides were seen to nucleate on the surface exposed after spallation. Elemental mapping revealed these newly nucleated oxides were rich in Cr.

After prolonged oxidation at 1050 °C for 50 h, severe oxidation occurred, leading to the complete detachment of the oxide layer. The SEM micrographs revealed the formation of stress-induced cracks extending into the metal subsurface, which developed due to severe oxidation and associated thermal stresses. Upon continuing oxidation cycles, failure of the protective oxide layer is expected to occur more rapidly at these cracked regions [44].

### 3.4. Oxide Layer Distribution and Its Thickness by Cross-Section Imaging and Elemental Mapping

**Figure 7:**
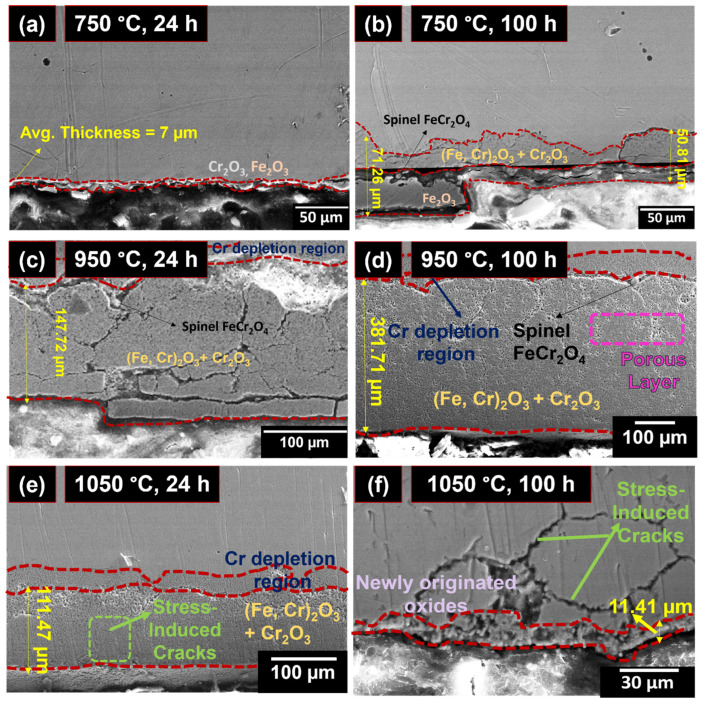
Cross-sectional micrographs showing (**a**) initial stage of oxidation where a thin oxide film originates at the metal subsurface; (**b**) dense oxide layer containing a duplex arrangement with an outer Fe_2_O_3_ and an inner Cr_2_O_3_ and (Fe, Cr)_2_O_3_ layer, with FeCr_2_O_4_ spinels; (**c**) region of minor spallation where the Fe_2_O_3_ layer has spalled but the inner oxide layer remains dense; (**d**) major spallation stage where the inner oxide layer becomes porous and a Cr-depleted zone develops beneath it; (**e**) minor spallation at 1050 °C where the inner mixed oxide layer and FeCr_2_O_4_ spinel remain intact; (**f**) major oxide spallation and the regeneration of new oxides in the alloy subsurface.

The thickness of the oxide scale and the kinetics of oxide scale evolution were analyzed using cross-sectional micrographs (Figure 7) and spot EDS analysis (Table 2). Nucleation of oxides was seen in the cross-sectional micrographs (Figure 7a) as a thin oxide layer during oxidation for 24 h at 750 °C. The oxide layer’s composition was difficult to ascertain due to the reduced oxide scale thickness. The average thickness of the oxide layer was approximately 7 µm. Also, the oxide layer thickness increased with an increase in exposure period. Fe_2_O_3_ was found to be the outer oxide layer, while a mixture of Cr_2_O_3_, (Fe, Cr)_2_O_3_ and spinel-type FeCr_2_O_4_ oxides makes up the inner oxide layer. These layers were found to be pore-free and uniformly distributed along the subsurface. The average oxide thickness was approximately 50.81 µm in the regions where the Fe_2_O_3_ layer was absent and increased to about 71.26 µm in the regions where both Fe_2_O_3_ and mixed oxide layers were present.

At 950 °C, both minor spallation and major spallation of the oxide layer were observed in the cross-sectional images after 24 and 50 h of isothermal oxidation. After 24 h, micrographs were taken from the regions exhibiting minor spallation, which revealed the absence of the Fe_2_O_3_ outer layer. However, the underlying mixed oxide layer remained dense and uniform in these regions, indicating that the oxide scale retained its protective nature despite the premature spallation of the Fe_2_O_3_ layer. The observed spallation was negligible and did not significantly affect the oxidation kinetics of the system. At this stage, the average thickness of the oxide layer was approximately 147.72 µm. After 50 h of oxidation, the inner oxide scale became porous and loose. The diffusion of oxygen ions through these pores into the metal matrix increased, leading to a breakdown oxidation stage characterized by complete detachment of the oxide layer. Furthermore, a Cr-depleted layer was observed beneath the inner oxide scale, where the chromium concentration was significantly reduced, as confirmed by the spot EDS analysis (point 3 in Figure 7d).

Cross-sectional micrographs of the oxidized samples for 1 h and 24 h exposure are presented in Figure 7e and Figure 7f, respectively. The oxide layers formed under these conditions were similar to those observed after oxidation at 950 °C for 50 h. Oxidation during 1 h of exposure at 1050 °C was considerably more severe than that observed after 24 h of oxidation at 750 and 950 °C, despite the shorter exposure duration. Pronounced spallation of the outer Fe_2_O_3_ layer and coarsening of the inner Fe Cr_2_O_4_ spinel layer were evident. Spot EDS analyses performed at points 4 and 5 revealed the presence of a chromium-depleted region beneath the oxide scale, along with a Cr- and Fe-rich mixed oxide layer. Meanwhile, minor stress-induced microcracks were found in the inner oxide layer, indicating the degradation of the oxide scale integrity at elevated temperatures.

After 24 h of oxidation at 1050 °C, spallation of both the Fe_2_O_3_ outer layer and the mixed oxide layer was observed. These stress-induced cracks became wider and penetrated deeper into the metal matrix compared to the sample oxidized for 1 h. Furthermore, the formation of a new oxide layer in the alloy subsurface was observed following the complete spallation of the previously formed oxide layers. Spot EDS analysis confirmed that this newly formed oxide layer was rich in Fe and Cr oxides.

### 3.5. Oxidation Mechanism of 08X14H SS in an Air Environment

**Figure 8:**
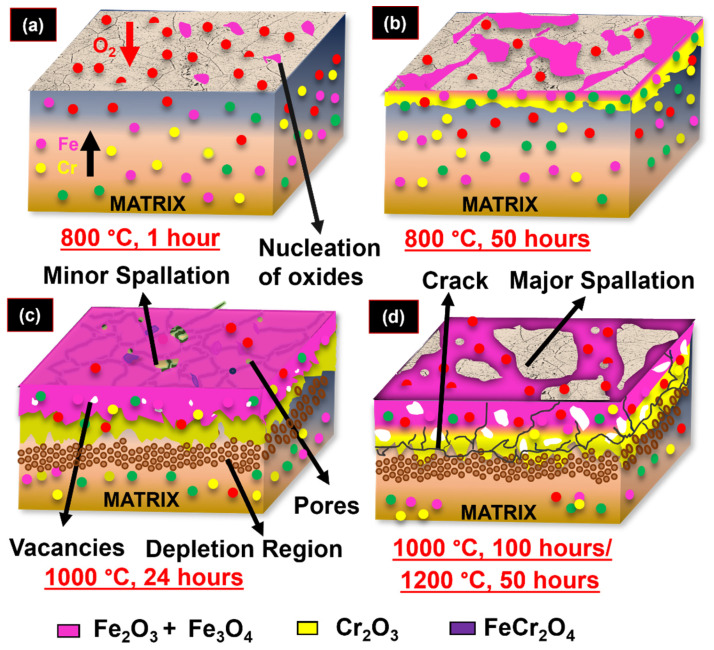
The schematic representation showing (**a**) initial oxidation stage where oxides nucleate [The red arrow represents the inward difussion of oxygen ions and black arrow represents the outward difussion of Fe and Cr ions during oxidation with respect to time and exposure period]; (**b**) the development of the discontinuous oxide layer; (**c**) development of voids, pores and oxide blisters on the oxide layers; and (**d**) major spallation of the oxide scale from the alloy subsurface.

The outward diffusion of metallic elements and the inward diffusion of oxygen control the oxidation of 08X14H steel in an air environment. During the initial stages, oxidation proceeds rapidly because the bare metal surface is directly exposed to the atmosphere, promoting faster diffusion of both oxygen and metal ions. This leads to the nucleation of Fe_2_O_3_ and Cr_2_O_3_ oxides at the alloy–gas interface (Figure 8a). The preferential formation of these oxides occurs because they possess the lowest dissociation oxygen partial pressures among the alloying elements. The nucleated oxides progressively grow across the surface and coalesce to form a continuous oxide layer (Figure 8b). Beneath the outer Fe_2_O_3_ layer, a continuous inner layer of Cr_2_O_3_ and (Cr, Fe)_2_O_3_ with FeCr_2_O_4_ spinel was observed. Both weight gain and oxide layer thickness increased with exposure period, while the oxidation rate progressively decreased. This behavior is attributed to the initially formed oxide layer, which functions as a diffusion barrier, limiting the transit of metallic species and oxygen while insulating the material surface from the external environment. Consequently, the rate of weight gain decreases, as reflected in the weight gain curves (Figure 1), and the oxide layer grows more slowly during subsequent oxidation periods. These oxide layers were dense, and their thickness increased with increasing temperature and exposure duration.

A similar oxidation mechanism was observed at 950 and 1050 °C; however, the rate of oxide formation was significantly higher, and the oxide phases appeared at much shorter exposure times. At these elevated temperatures, severe oxidation accelerated the entire process, ultimately leading to material failure (observed during exposure up to 100 h at 950 °C and 50 h at 1050 °C) (Figure 8d). The rapid progression of oxidation at high temperatures caused the protective oxide scale to lose its protective nature, resulting in spallation or complete detachment of the scale from the surface. The loss of protective behavior is attributed to oxide coarsening, which generates voids and is considered the initial step toward oxide scale degradation (Figure 8c). Subsequently, the oxide layer becomes increasingly porous and unstable, allowing selective internal oxidation along grain boundaries. This internal oxidation promotes the formation of FeCr_2_O_4_ spinels, further reducing the oxidation resistance of the material.

The combined effect of void formation and spinel development increases the stress within the oxide scale, leading to stress-induced cracking, which is clearly visible in cross-section micrographs of samples exhibiting major or minor spallation at 950 and 1050 °C. A chromium-depleted zone was identified beneath the duplex oxide scale, with its thickness increasing at critical stages (1050 °C, 1 h). The above-mentioned stage corresponds to the breakdown stage, where the rate of oxidation, weight gain, and oxide layer thickness increase sharply, ultimately resulting in complete detachment of the scale. At the sites where the spallation occurred, renewed nucleation of the fresh oxide nodules was evident.

## 4. Conclusions

In the present study, the isothermal oxidation behavior of heat-treated 08X14H SS was investigated in an air environment at 750, 950 and 1050 °C for exposure durations up to 100 h. Weight gain curves revealed the oxidation kinetics, which followed a parabolic rate law regardless of the exposure conditions, indicating diffusion-controlled oxidation behavior. Characterization techniques, including XRD, surface and cross-sectional FESEM analyses, and elemental mapping, were used to evaluate the oxide scale morphology, elemental distribution, and oxidation kinetics of 08X14H stainless steel in air. The oxide scale primarily consisted of Fe_2_O_3_, (Fe, Cr)_2_O_3_, Cr_2_O_3_ and FeCr_2_O_4_ spinel phases. The oxide layer comprised an outer Fe_2_O_3_ layer and an inner Cr_2_O_3_ and (Fe, Cr)_2_O_3_ layer with embedded FeCr_2_O_4_ spinels. Under severe oxidation conditions, the oxide scale became loose and exhibited stress-induced cracks and voids. Significant spallation of the external Fe-rich oxides was observed, ultimately leading to material degradation. In an air environment, major oxide scale spallation occurred after oxidation for 100 h at 950 °C and 50 h and 1050 °C. Based on these findings, the newly developed 08X14H is recommended for long-term applications up to 100 h at 750 °C. However, at high temperatures of 950 and 1050 °C, its application should be limited to short-term exposure of up to 50 h and 24 h, respectively, in air.

## Figures and Tables

**Table 1:** Chemical composition of base 08X14H SS.

Element	Cr	Ni	Mo	Si	Mn	Cu	C	P	S	Fe
Composition (wt.%)	14.85	8.5	0.95	0.6	0.6	0.3	0.08	0.03	0.03	Bal

**Table 2:** Spot EDS analysis at selected points marked in Figure 7, showing the elemental composition (at.%).

Points	Elemental Composition (at.%)				
	Fe	Cr	Ni	Mn	O
1	39.67	6.01	3.89	0.30	32.68
2	17.23	12.14	0.29	0.32	44.54
3	33.11	6.44	4.48	0.24	23.37
4	19.21	2.71	2.37	0.32	30.59
5	3.76	1.74	0.06	0.29	34.58
6	32.18	3.78	7.71	0.18	44.04
7	60.90	12.73	5.19	0.92	2.79

## Data Availability

The original contributions presented in this study are included in the article. Further inquiries can be directed to the corresponding author.
